# Culling reasons and risk factors in Estonian dairy cows

**DOI:** 10.1186/s12917-020-02384-6

**Published:** 2020-06-01

**Authors:** Triin Rilanto, Kaari Reimus, Toomas Orro, Ulf Emanuelson, Arvo Viltrop, Kerli Mõtus

**Affiliations:** 1 Institute of Veterinary Medicine and Animal Sciences, Estonian University of Life Science, Kreutzwaldi 62, 51006 Tartu, Estonia; 2 Department of Clinical Sciences, Swedish University of Agricultural Sciences, SE-75007 Uppsala, Sweden

**Keywords:** Dairy cow, Estonia, Culling, Slaughter

## Abstract

**Background:**

Culling is a major cost for dairy farms but also an essential part in managing herd productivity. This study aimed to identify the culling rates of Estonian dairy cows, identify the farmers’ stated reasons and risk factors for culling. This observational study used registry data of all cows from herds with ≥20 cow-years in 2013–2015. Cow lactation-level analyses included data of 86,373 primiparous cows from 409 herds and 177,561 lactations of 109,295 multiparous cows from 410 herds. Weibull proportional hazard regression models were used to identify risk factors for culling due to slaughter or death.

**Results:**

The overall culling rate of Estonian dairy cows was 26.24 (95% CI 26.02; 26.46) per 100 cow-years. The most common reasons farmers stated for culling were feet/claw disorders (26.4%), udder disorders (22.6%), metabolic and digestive disorders (18.1%) and fertility problems (12.5%). Animal-level risk factors for culling were Holstein breed, older parity, lower milk yield breeding value, older age at first calving, longer previous calving interval, having assisted calving, stillbirth and birth of twins/triplets. Lower milk yield, somatic cell count over 200,000 cells/ml and fat/protein ratio over 1.5 at first test-milking after calving were associated with greater culling hazard during the lactation. Cows from larger herds, herds with decreasing size and higher milk yields had a higher culling probability.

**Conclusions:**

This study emphasises the need for improved management of hoof health and prevention of mastitis and metabolic diseases. It is essential to ensure easy calving and good health of cows around calving in order to lower the culling hazard.

## Background

Culling is defined as the departure of cows from the herd because of sale, slaughter, salvage, or death [1]. Culling is an important cost for dairy farms [1–3]. At the same time, culling is a way to increase herd productivity and profitability, as keeping diseased and unproductive cows might result in lower herd milk production and deteriorated reproduction. Keeping cows too long in a herd might also impair the herd’s genetic improvement [3]. In order to maximize profitability, the proportion of voluntary culling (selling for dairy purposes or culling due to low production) should be highest among the total culling rate [1, 3, 4]. Previous studies indicate an ascending trend in the proportion of involuntary culling [4, 5]. Culling rate, especially the proportion of involuntary culls, may also be considered as an animal welfare indicator [4, 6, 7].

During the last decades, culling rates have not increased; however, the longevity of cows has declined worldwide [8, 9]. In Estonia, the average productive age of culled cows has decreased from 1113 to 1051 days between 2013 and 2018, respectively [10, 11]. Longer productive lifetime would result in lower replacement costs, the opportunity to sell heifers [12, 13] and the possibility of increasing the proportion of voluntary culling, bearing a desirable effect on herd profitability [14]. Return of the rearing costs up to first calving are covered roughly by the start of the second lactation [13, 15]. Milk yield reaches the highest level after the third lactation [11], meaning that culling young cows is especially undesirable. Importantly, short lifespan is against consumers’ expectations and is also associated with more detrimental environmental impacts [16]. Culling reasons have changed over the last decades, referring to the proportion of culling due to voluntary reasons (e.g., low milk yield), which has decreased and shifted into rather disease-related reasons [9].

Estonia is located in Northern Europe on the coast of Baltic Sea. Having a temperate climate condition the average temperature is + 5 C° being − 4 … -5 C° in winter months and 15 … 18 C° in summer months. Estonian dairy cow population constitutes approximately 85,000 dairy cows, with roughly 67% of the dairy cows being housed in farms with more than 300 cows in 2018 (Estonian Agricultural Registers and Information Board, 2018). The average milk yield of Estonian dairy cows was 9785 kg in 2018 (Estonian Livestock Performance Recording Yearbook, 2018), taking second place in the EU (Eurostat, 2019). The majority of large Estonian dairy farms are loose-housed open-air barns using modern equipment and technology managed by hired labour. The Estonian dairy cow population is endemically infected with many cattle pathogens, including bovine herpesvirus 1, bovine respiratory syncytial virus and bovine viral diarrhea virus [17], digital dermatitis [18] as well as with main contagious mastitis pathogens *Staphylococcus aureus, Streptococcus agalactiae* and *Mycoplasma bovis* [19]. Due to these population characteristics, the results of previous studies investigating reasons and risk factors for dairy cow culling might not apply to these herds. Due to worldwide intensification of dairy production [20], the Estonian dairy cow population could serve as a valuable example, representing herds with high production level and mostly intensive keeping conditions.

In order to make use of opportunities that may be afforded by increasing cow longevity, there is a need for an overview about the main reasons as well as the risk factors that influence the probability of culling. This information would allow for the establishment of better-focused hypotheses for further studies that aim to reveal causal associations between animal and herd-level risk factors and dairy cow longevity. The aim of the present study was to analyse the culling rates of Estonian dairy cows, identify the frequency of farmers’ stated reasons for dairy cow culling, and investigate risk factors for cow culling.

## Results

### Descriptive statistics

**Fig. 1:**
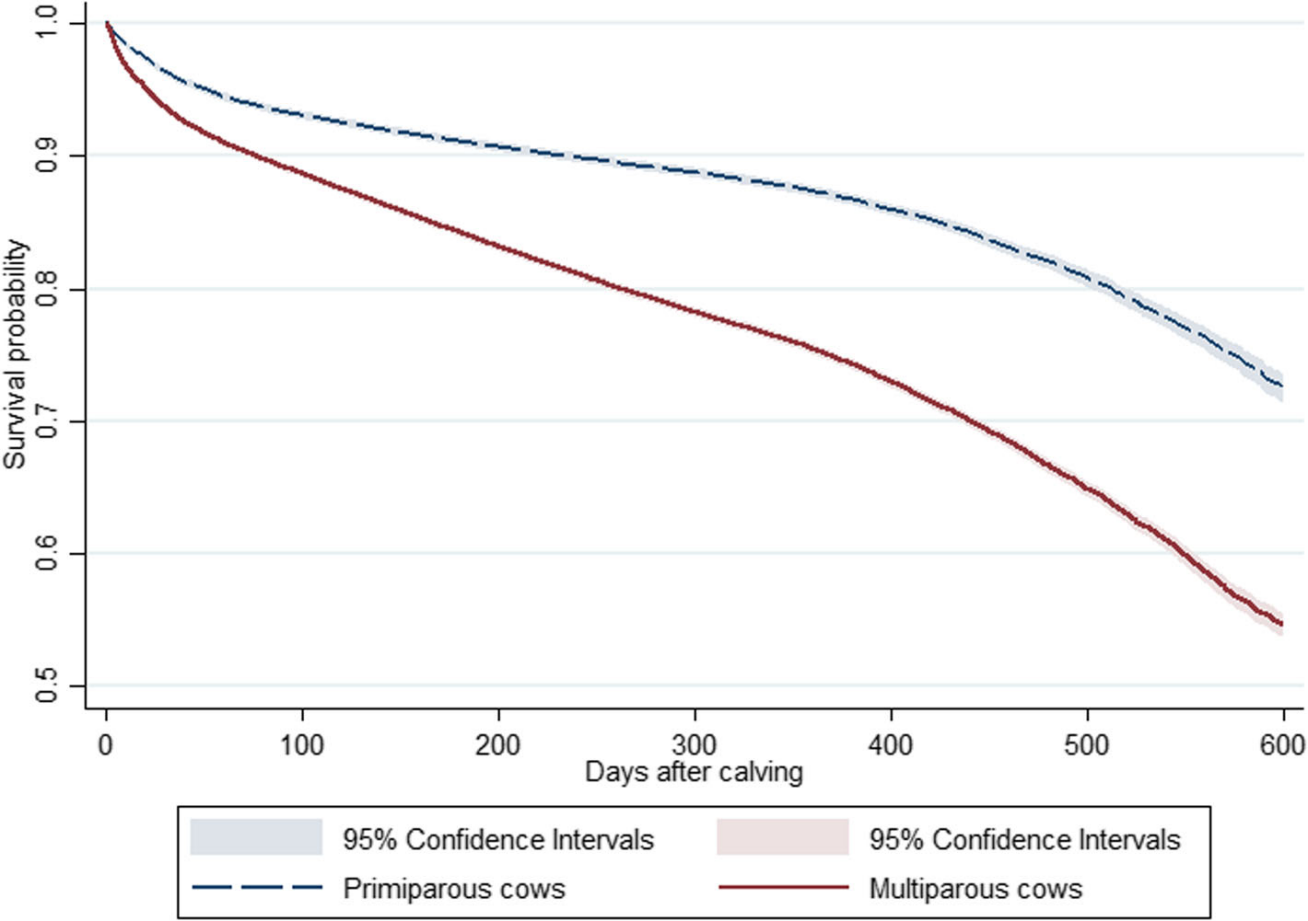
Kaplan-Meier survival curve for Estonian primiparous and multiparous cows followed from calving to culling (on-farm death, euthanasia or slaughter) or right censoring in 86,373 primiparous and 109,295 multiparous cows in the period between January 1, 2013 to December 31, 2015

The final datasets used for the analyses included the data of 263,934 lactations of 154,057 cows reared in 410 herds. In the dataset, 71,442 (46.4%) cows had a single lactation, 55,689 (36.2%) cows had two lactations, 26,590 (17.2%) cows had three, and 336 (0.2%) cows had four lactations, whereas 67,684 primiparous cows were repeated in the multiparous cow dataset. In total, 86,373 lactation level records of primiparous cows reared in 409 herds, and 177,561 lactations of 109,295 multiparous cows from 410 herds were analysed separately. The overall culling rate (**CR**) of Estonian dairy cows was 26.24 (95% CI 26.02; 26.46) per 100 cow-years. Among primiparous cows 3348 (3.9%) died on-farm, 95 (0.1%) were euthanized, 8044 (9.3%) cows were transported to slaughter, 3544 (4.1%) were sold to another herd and four were lost during the lactation level observation period resulting in a CR of 15.95 (95% CI 15.66; 16.24) per 100 cow-years. In the multiparous cow dataset, there were 10,756 (6.1%) death records, 260 (0.2%) cows were euthanized, 31,248 (17.6%) were transported to slaughter, 12,144 (6.8%) were sold and 25 cows were lost. The CR was 31.83 (95% CI 31.52; 32.12) per 100 cow-years among multiparous cows. The hazard of culling was significantly higher in multiparous cows compared to primiparous cows in the univariable Weibull random-effect model (hazard rate ratio (**HR**) = 2.04, 95% CI 2.00; 2.08, *p* < 0.001) (Fig. 1).

**Fig. 2:**
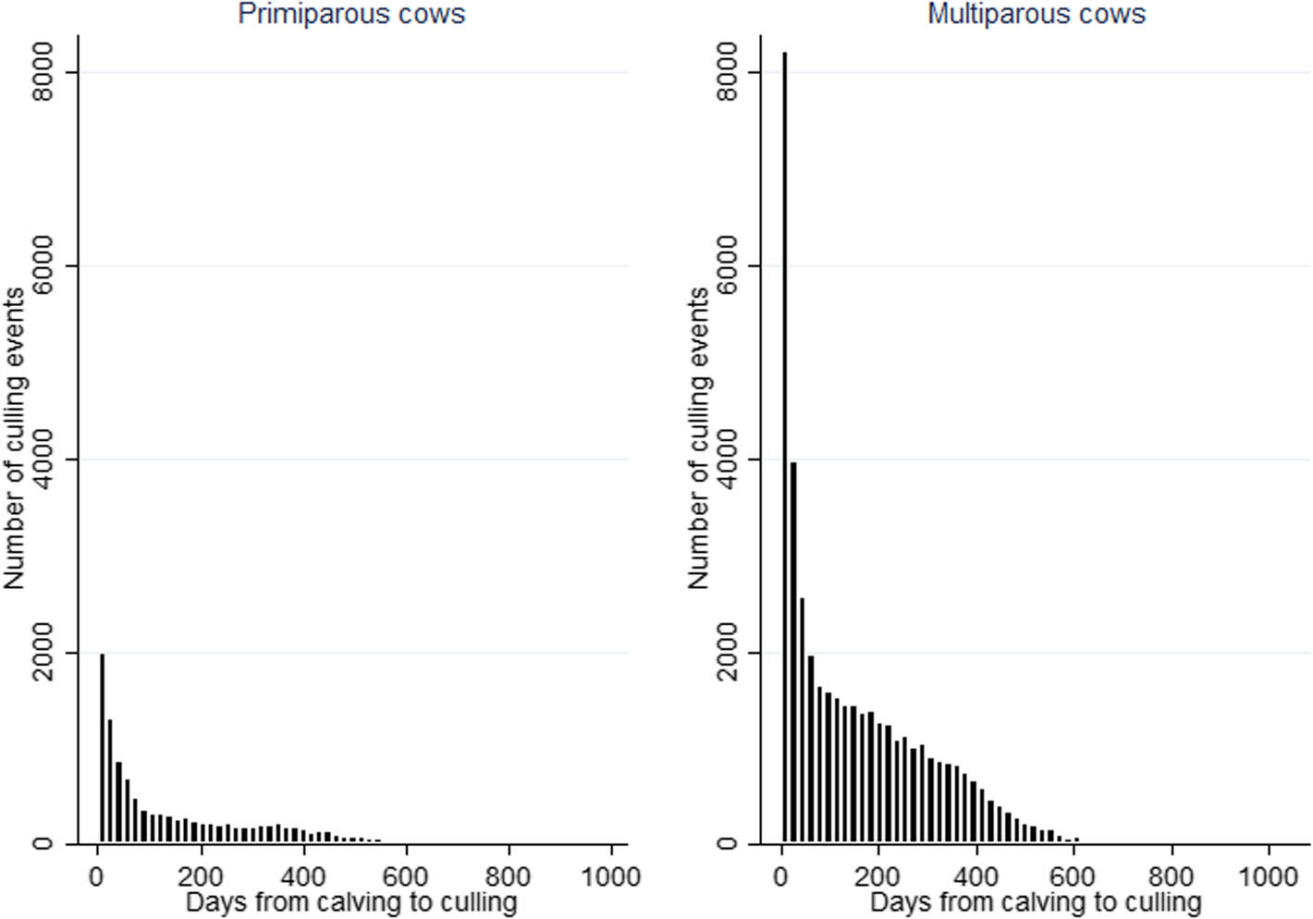
Distribution of culling events (on-farm mortality, euthanasia or slaughter) over lactation in 11,491 Estonian primiparous and 42,289 multiparous culled cows between January 1, 2013 to December 31, 2015

The culling risk was highest during the early post-partum period (Figs. 1 and 2). By 30 days after calving the survivor probability of primiparous cows was 0.96 (95% CI 0.96; 0.97) and 0.94 (95% CI 0.94; 0.94) in multiparous cows. The average survival probability was 4.42% lower in multiparous cows compared to primiparous cows at 100 days of lactation. The survival probability dropped from 0.91 (95% CI 0.91; 0.91) to 0.89 (95% CI 0.88; 0.89) in primiparous cows and from 0.83 (95% CI 0.83; 0.83) to 0.78 (95% CI 0.78; 0.78) in multiparous cows between 200 to 305 days after calving (**DAC**), respectively. The survival probability declined by 16.1 and 23.5% between 305 and 600 DAC in primiparous and multiparous cows, respectively (Fig. 1). Around one year after calving, a small increase in the number of culling events was observed among primiparous cows and a stabilisation of continuous decrease of number of culling events over lactation was also identified in multiparous cows at that time (Fig. 2).

### Farmers’ stated reasons for culling

Culling reason was missing for 95 and 366 culling events in primiparous and multiparous cows, respectively. Main causes of culling due to death and slaughter were reported as feet/claw disorders (26.4% of culled cows), udder disorders (22.6%), metabolic and digestive disorders (18.1%), and fertility problems (12.5%). The importance of fertility problems as the reason for culling decreased after the second parity (Table 1).

**Table 1:** Distribution of farmers´ stated reasons for Estonian culled dairy cows due to death and slaughter over parities in years 2013 to 2015

	Parity						
Reason of culling	1 (%)^a^	2 (%)^a^	3 (%)^a^	4 (%)^a^	5 (%)^a^	≥6 (%)^a^	Total (%)^a^
Feet/claw disorders	2875 (25.2)	2953 (26.3)	2913 (27.1)	2355 (27.3)	1499 (26.6)	1487 (26.2)	14,082 (26.4)
Respiratory and infectious diseases	208 (1.8)	168 (1.5)	139 (1.3)	77 (0.9)	46 (0.8)	29 (0.5)	667 (1.3)
Metabolic and digestive disorders	1527 (13.4)	1932 (17.2)	2193 (20.4)	1855 (21.5)	1197 (21.2)	950 (16.8)	9654 (18.1)
Fertility problems	1736 (15.2)	1782 (15.9)	1322 (12.3)	865 (10.0)	519 (9.2)	457 (8.1)	6681 (12.5)
Dystocia	561 (4.9)	232 (2.1)	211 (2.0)	185 (2.2)	142 (2.5)	116 (2.1)	1447 (2.7)
Trauma and accident	615 (5.4)	488 (4.3)	403 (3.7)	266 (3.1)	157 (2.8)	138 (2.4)	2067 (3.9)
Udder disorders	2046 (18.0)	2404 (21.4)	2569 (23.9)	2157 (25.0)	1455 (25.8)	1418 (25.0)	12,049 (22.6)
Low milk yield	930 (8.2)	525 (4.7)	378 (3.5)	276 (3.2)	175 (3.1)	168 (3.0)	2452 (4.6)
Age	1 (0.01)	2 (0.02)	9 (0.1)	38 (0.4)	92 (1.6)	552 (9.7)	694 (1.3)
Other^b^	897 (7.9)	749 (6.7)	630 (5.9)	540 (6.3)	359 (6.4)	351 (6.2)	3526 (6.6)
Total	11,396 (21.4)^c^	11,235 (21.1)^c^	10,767 (20.2)^c^	8614 (16.2)^c^	5641 (10.6)^c^	5666 (10.6)^c^	53,319 (100.0)

^a^ n (% of total n culled in respective parity)

^b^animal lost, bad temperament, bad milking, selling, other reasons

^c^ n (% of total n culled)

In total, 50.7 and 46.5% of the culling events occurred during the first 100 DAC in primiparous and multiparous cows, respectively. Roughly one-third (33.9%) of the cullings took place at more than 200 DAC. Metabolic and digestive disorders were more prevalent up to 100 DAC (17.1 and 31.7% in primiparous and multiparous cows, respectively) compared to later stages of lactation (11.5 and 11.1% in primiparous and multiparous cows, respectively in 101–200 DAC). Feet and claw disorders (30.9 and 33.1% in primiparous and multiparous cows, respectively) and udder disorders (25.0 and 35.0% in primiparous and multiparous cows, respectively) were most common reasons for culling in the middle of the lactation period. Fertility problems were the primary reasons for culling in the late lactation stage (≥200 DAC), comprising 38.2 and 28.1% of all culls among primiparous and multiparous cows, respectively. Across all culling reasons, metabolic disorders were nearly twice as frequently the cause of culling in multiparous cows compared to primiparous cows. In primiparous cows, dystocia and low milk yield both constituted roughly 9% of all culling reasons during the first 100 DAC, being higher than that reported in multiparous cows (4.1 and 2.0%, respectively) (Table 2).

**Table 2:** Distribution of farmers´ stated reasons for Estonian culled dairy cows due to death and slaughter over lactation stages in years 2013 to 2015

	Primiparous cows			Multiparous cows		
Reason of culling	< 100 DAC^a,b^	101–200 DAC^a,b^	≥200 DAC^a,b^	< 100 DAC^a,b^	101–200 DAC^a,b^	≥200 DAC^a,b^
Feet/claw disorders	1575 (27.2)	539 (30.9)	761 (19.7)	4990 (25.6)	2721 (33.1)	3496 (24.6)
Respiratory and infectious diseases	95 (1.6)	44 (2.5)	69 (1.8)	258 (1.3)	93 (1.1)	108 (0.8)
Metabolic and digestive disorders	991 (17.1)	200 (11.5)	336 (8.7)	6177 (31.7)	913 (11.1)	1037 (7.3)
Fertility problems	173 (3.0)	85 (4.9)	1478 (38.2)	615 (3.2)	332 (4.0)	3998 (28.1)
Dystocia	520 (9.0)	2 (0.1)	39 (1.0)	793 (4.1)	2 (0.02)	91 (0.6)
Trauma and accident	326 (5.6)	107 (6.1)	182 (4.7)	826 (4.2)	256 (3.2)	370 (2.6)
Udder disorders	1120 (19.4)	437 (25.0)	489 (12.7)	4140 (21.2)	2876 (35.0)	2987 (21.0)
Low milk yield	526 (9.1)	159 (9.1)	245 (6.4)	381 (2.0)	383 (4.7)	758 (5.3)
Age	0 (0)	0 (0)	1 (0.003)	133 (0.7)	140 (1.7)	420 (3.0)
Other^c^	456 (7.9)	174 (10.0)	267 (6.9)	1181 (6.0)	494 (6.0)	954 (6.7)
Total	5782 (50.7)^d^	1747 (15.3)^d^	3867 (33.9)^4^	19,494 (46.5)^e^	8210 (19.6)^e^	14,219 (33.9)^e^

^a^DAC - days after calving

^b^ n (% of total n culled during specific lactation stage)

^c^animal lost, bad temperament, bad milking, selling, other reasons

^d^proportion of primiparous cows culled during specific lactation stage

^e^proportion of multiparous cows culled during specific lactation stage

### Animal-level risk factors for culling

Descriptive statistics and univariable associations between continuous and categorical predictor variables and culling are presented in Supplementary Tables 1A and 1B, respectively.

Several culling risk factors were common for primiparous and multiparous cows. Estonian Red and Estonian Native breed cows had significantly lower culling hazard compared to Estonian Holstein breed cows (HR = 0.92, 95% CI 0.85; 0.98 and HR = 0.84, 95% CI 0.81; 0.87 in primiparous and multiparous cows, respectively). Higher milk yield breeding value was a protective factor for culling. In primiparous cows, the culling hazard was significantly lower in 2015 (HR = 0.90, 95% CI 0.85; 0.94) compared to 2013. In multiparous cows, the culling hazard was significantly higher in 2014 (HR = 1.08, 95% CI 1.06; 1.10) and 2015 (HR = 1.05, 95% CI 1.02; 1.07) compared to 2013. Having a stillborn calf (HR = 1.45, 95% CI 1.37; 1.54 and HR = 1.74, 95% CI 1.68; 1.81 in primiparous and multiparous cows, respectively), abortion (HR = 2.69, 95% CI 2.34; 3.10 in multiparous cows) or twins/triplets (HR = 1.31, 95% CI 1.05; 1.63 and HR = 1.35, 95% CI 1.29; 1.42 in primiparous and multiparous cows, respectively) were associated with a higher risk for culling compared to giving birth to a single female calf. In multiparous cows, the culling hazard was also higher in cows that gave birth to a male calf compared to a female calf (HR = 1.05, 95% CI 1.03; 1.07). There was a time-dependent effect of assisted calving to culling hazard – the negative effect of assisted calving on culling hazard was higher during the first seven days post-partum compared to a later period. Also, higher age at first calving was associated with a higher culling hazard (HR = 1.07, 95% CI 1.06; 1.07 and HR = 1.02, 95% CI 1.02; 1.03 in primiparous and multiparous cows, respectively) but the association was not linear (Tables 3 and 4).

**Table 3:** Results of multivariable random-effect Weibull model for risk factors of culling in 85,765 primiparous dairy cows (herds *n* = 389)

Variable	Category	N^a,b^	Hazard Rate Ratio	95% Confidence Intervals	P-value	Likelihood ratio test p-value
*Animal level variables*						
Breed^a^	Estonian Holstein	70,508	1			0.018
	Estonian Red and Estonian Native	15,257	0.92	0.85; 0.98	0.018	
Relative milk yield breeding value^a^	< 90	17,622	1			< 0.001
	90–96	20,022	0.66	0.63; 0.71	< 0.001	
	97–104	18,065	0.48	0.45; 0.52	< 0.001	
	≥105	21,320	0.37	0.35; 0.40	< 0.001	
	Not estimated	8736	14.50	13.74; 15.31	< 0.001	
Calving year^a^	2013	27,904	1			< 0.001
	2014	29,368	1.01	0.97; 1.06	0.536	
	2015	28,493	0.90	0.85; 0.94	< 0.001	
Calf^a^	Female	38,482	1			< 0.001
	Male	36,502	1.04	0.996; 1.081	0.080	
	Stillbirth	9972	1.45	1.37; 1.54	< 0.001	
	Twins/triplets	480	1.31	1.05; 1.63	0.015	
	Abortion	329	0.67	0.50; 0.91	0.011	
Assisted calving x period^b^	No, < 7 days	70,351	1			< 0.001
	No, > 7 days	69,176	1.13	1.04; 1.23	0.004	
	Yes, < 7 days	15,414	2.10	1.81; 2.36	< 0.001	
	Yes, > 7 days	14,939	1.34	1.22; 1.48	< 0.001	
Age at first calving (months) (centered)			1.07	1.06; 1.07	< 0.001	< 0.001
Square term centered value of age at first calving (months)			0.99	0.994; 0.995	< 0.001	< 0.001
*Herd-level variables*						
Change of herd size from 2013 to 2015^a^	No change (±5%)	39,143	1			< 0.001
	Decrease > 5 to 15%	12,904	1.28	1.001; 1.631	0.049	
	Decrease > 15%	8499	1.56	1.22; 2.00	< 0.001	
	Increase > 5 to 15%	13,591	0.79	0.62; 1.01	0.060	
	Increase > 15%	11,628	0.61	0.47; 0.80	< 0.001	
Herd average number of cows (increase by 50 cows)			1.04	1.02; 1.05	< 0.001	< 0.001
Herd average interval from calving to insemination (days)			0.99	0.985; 0.992	< 0.001	< 0.001
Herd average first insemination conception rate (%)			1.01	1.01; 1.02	< 0.001	< 0.001
Herd average number of lactations (years)			0.41	0.33; 0.51	< 0.001	< 0.001
Region^a,c^	North-East	36,651	1			0.018
	South-East	17,235	0.85	0.71; 1.02	0.086	
	South-West	19,091	1.14	0.98; 1.32	0.087	
	North-West	12,788	1.04	0.87; 1.24	0.665	

Shape parameter *p* = 0.82

^a^number of animals in each category

^b^number of observations in each category after splitting the observations in 7th day post-calving

^c^Northeast Estonia: Ida-Viru, Lääne-Viru, Jõgeva, Järva county; Southeast Estonia: Tartu, Valga, Võru, Põlva county; Southwest Estonia: Pärnu, Viljandi, Saare county; Northwest Estonia: Harju, Rapla, Lääne, Hiiu county

**Table 4:** Results of multivariable random-effect Weibull model for risk factors of culling in 107,835 multiparous dairy cows in 173,773 lactations (herds *n* = 409)

Variable	Category	N^a,b^	Hazard Rate Ratio	95% Confidence Intervals	P-value	Likelihood ratio test p-value
*Animal level variables*						
Breed^a^	Estonian Holstein	85,879	1			
	Estonian Red and Estonian Native	21,956	0.84	0.81; 0.87	< 0.001	< 0.001
Relative milk yield breeding value^a^	< 90	23,731	1			< 0.001
	90–96	23,607	0.97	0.94; 0.997	0.028	
	97–104	27,319	0.94	0.91; 0.97	< 0.001	
	≥105	27,704	0.88	0.86; 0.91	< 0.001	
	Not estimated	5474	0.94	0.88; 0.99	< 0.001	
Calving year^b^	2013	56,644	1			< 0.001
	2014	58,792	1.08	1.06; 1.10	< 0.001	
	2015	58,337	1.05	1.02; 1.07	0.001	
Abortion in previous lactation^b^	No	172,949	1			
	Yes	824	0.86	0.76; 0.98	0.025	0.025
Previous calving interval (increase of ten days, in months) (centered)			1.03	1.03; 1.04	< 0.001	< 0.001
Square term centered value of previous calving interval (increase of ten days, in months) (centered)			0.99998	0.99983; 0.99994	< 0.001	< 0.001
Parity^b^	Second	66,191	1			< 0.001
	Third	46,171	1.38	1.34; 1.42	< 0.001	
	Fourth	29,511	1.81	1.76; 1.87	< 0.001	
	Fifth	16,868	2.22	2.14; 2.29	< 0.001	
	Sixth	8566	2.49	2.39; 2.60	< 0.001	
	≥Seventh	6466	3.13	2.99; 3.28	< 0.001	
Calf^b^	Female	75,311	1			< 0.001
	Male	82,247	1.05	1.03; 1.07	< 0.001	
	Stillbirth	9063	1.74	1.68; 1.81	< 0.001	
	Twins/triplets	6655	1.35	1.29; 1.42	< 0.001	
	Abortion	497	2.69	2.34; 3.10	< 0.001	
Assisted calving x period^c^	No, < 7 days	159,057	1			< 0.001
	No, > 7 days	154,449	0.51	0.49; 0.53	< 0.001	
	Yes, < 7 days	14,670	2.34	2.16; 2.55	< 0.001	
	Yes, > 7 days	13,838	0.59	0.56; 0.62	< 0.001	
Milk somatic cell count at last test-milking in previous lactation (*1000/ml)^b^	< 200		1			
	≥200		1.21	1.18; 1.23	< 0.001	< 0.001
Days in milk during last test-milking in previous lactation (days)^b^			0.998	0.9978; 0.998	< 0.001	< 0.001
Milk yield at last test-milking in previous lactation (kg)^b^			0.99	0.989; 0.99	< 0.001	< 0.001
Milking method at last test-milking of previous lactation^b^	Milking twice a day		1			< 0.001
	Milking three times a day		0.96	0.89; 1.04	0.303	
	Robot milking		0.83	0.78; 0.89	< 0.001	
Age at first calving (months) (centered)			1.02	1.02; 1.03	< 0.001	< 0.001
Square term centered value of age at first calving (months)			0.9995	0.9991; 0.9999	0.024	0.024
*Herd level variables*						
Change of herd size from 2013 to 2015^a^	No change (±5%)	48,548	1			< 0.001
	Decrease > 5 to 15%	16,286	1.06	0.92; 1.21	0.444	
	Decrease > 15%	12,204	1.33	1.15; 1.54	< 0.001	
	Increase > 5 to 15%	16,922	0.89	0.77; 1.02	0.091	
	Increase > 15%	13,875	0.82	0.70; 0.95	0.008	
Herd average calving interval (increase by 10 days)			0.97	0.95; 0.98	< 0.001	< 0.001
Herd average number of cows (increase by 50 cows)			1.02	1.01; 1.03	0.002	0.002
Herd average milk yield (increase by 500 kg)			1.04	1.02; 1.06	< 0.001	< 0.001
Herd average number of lactations (years) (centered)			0.49	0.42; 0.57	< 0.001	< 0.001
Square term centered value of herd average number of lactations (years)			1.11	1.06; 1.17	< 0.001	< 0.001
Region^a,d^	North-East	43,015	1			< 0.001
	South-East	23,039	0.82	0.73; 0.93	0.001	
	South-West	25,206	0.82	0.74; 0.90	< 0.001	
	North-West	16,575	0.90	0.81; 1.01	0.076	

Shape parameter *p* = 0.83

^a^number of animals in each category

^b^number of observations

^c^number of observations in each category after splitting the observations at 7th day post-calving

^d^Northeast Estonia: Ida-Viru, Lääne-Viru, Jõgeva, Järva county; Southeast Estonia: Tartu, Valga, Võru, Põlva county; Southwest Estonia: Pärnu, Viljandi, Saare county; Northwest Estonia: Harju, Rapla, Lääne, Hiiu county

In multiparous cows, the culling hazard increased with parity. Somatic cell count (**SCC**) over 200,000 per ml and lower milk yield at last test-milking before dry-off were factors associated with a higher culling hazard during the following lactation (HR = 1.21, 95% CI 1.18; 1.23 and HR = 0.99, 95% CI 0.989; 0.99, respectively). Robot milking system at last test-milking before dry-off was a protective factor for culling (HR = 0.83, 95% CI 0.78; 0.89) compared to other milking methods (Tables 3 and 4).

In addition, lower milk yield (HR = 0.96, 95% CI 0.956; 0.958), somatic cell count ≥200,000 cells/ml (HR = 1.31, 95% CI 1.28; 1.34) and milk fat/protein ratio ≥ 1.5 (HR = 1.23, 95% CI 1.20; 1.26) in first test-milking after calving were associated with a higher culling risk during the lactation. Using automatic milking system or milking cows three times a day at first test-milking were protective for cow culling probability during the lactation compared to milking cows twice a day (HR = 0.57, 95% CI 0.53; 0.61 and HR = 0.79, 95% CI 0.74; 0.84, respectively) (Table 5).

**Table 5:** Results of multivariable random-effect Weibull model for risk factors for culling due to slaughter and death in 232,445 lactations of 140,934 dairy cows (herds *n* = 410)

Variable	Category	N^a,b^	Hazard Rate Ratio	95% Confidence Intervals
*Animal-level variables*				
Days in milk at first test-milking (centered)			1.008	1.007; 1.009
Square term of days in milk at first test-milking (centered)			0.9997	0.9995; 0.9997
Milk yield at first test-milking (centered) (kg)			0.96	0.956; 0.958
Square term of milk yield at first test-milking (centered)			1.001	1.0011; 1.0013
Milk fat/protein ratio at first test-milking^b^	< 1.5	181,507	1	
	≥1.5	50,938	1.23	1.20; 1.26
Milk somatic cell count at first test-milking (*1000/mL)^b^	< 200	174,387	1	
	≥200	58,058	1.31	1.28; 1.34
Milking method at first test-milking^b^	Milking twice a day	163,033	1	
	Milking three times a day	41,773	0.79	0.74; 0.84
	Robot milking	27,639	0.57	0.53; 0.61
Breed^a^	Estonian Holstein	113,284	1	
	Estonian Red and Estonian Native	27,650	0.79	0.76; 0.82
Parity^b^	First	77,456	1	
	Second	60,689	2.09	2.03; 2.16
	Third	41,267	2.89	2.79; 2.99
	Fourth	25,810	3.65	3.52; 3.78
	Fifth	14,398	4.35	4.18; 4.53
	Sixth	7334	4.96	4.72; 5.21
	≥Seventh	5491	6.12	5.80; 6.45
Calving year^b^	2013	77,968	1	
	2014	81,011	0.996	0.97; 1.02
	2015	73,466	0.95	0.92; 0.97
*Herd-level variables*				
Herd average number of cows (one unit change is 50 cows)			1.01	1.001; 1.03
Herd average milk yield (one unit change is 500 kg)			1.15	1.13; 1.17
Region^a,d^	Northeast	117	1	
	Southeast	98	0.87	0.76; 0.9993
	Southwest	121	0.90	0.90; 1.003
	Northwest	74	0.90	0.80; 1.02
Change of herd size from 2013 to 2015^c^	No change (±5%)	135	1	
	Decrease > 5 to 15%	71	1.03	0.86; 1.23
	Decrease > 15%	68	1.48	1.24; 1.77
	Increase > 5 to 15%	74	0.89	0.74; 1.06
	Increase > 15%	62	0.86	0.71; 1.03

Shape parameter *p* = 1.09

^a^number of cows in each category

^b^number of observations in each category

^c^number of herds in each category

^d^Northeast Estonia: Ida-Viru, Lääne-Viru, Jõgeva, Järva county; Southeast Estonia: Tartu, Valga, Võru, Põlva county; Southwest Estonia: Pärnu, Viljandi, Saare county; Northwest Estonia: Harju, Rapla, Lääne, Hiiu county

### Herd-level factors associated with culling

Cow culling hazard was higher in herds that reduced their number of cows by more than 15% within the three study years (HR = 1.56, 95% CI 1.22; 2.00 and HR = 1.33, 95% CI 1.15; 1.54 compared to stable herd size in primiparous and multiparous cows, respectively), whereas expanding herd size (increase of number of cows > 15%) had a protective effect on culling (HR = 0.61, 95% CI 0.47; 0.80 and HR = 0.82, 95% CI 0.70; 0.95). On average, the culling hazard was higher in larger herds. Increase of herd size by 50 cows was associated with on average 4% higher culling hazard (HR = 1.04, 95% CI 1.02; 1.05) in primiparous cows and 2% (HR = 1.02, 95% CI 1.01; 1.03) higher culling hazard in multiparous cows (Tables 3 and 4).

The culling hazard was also negatively associated with herd average lactation number. Longer herd average interval from calving to insemination (HR = 0.99, 95% CI 0.985; 0.992) and longer herd average calving interval (HR = 0.97, 95% CI 0.95; 0.98) was associated with lower culling hazard in primiparous cows and multiparous cows, respectively (Tables 3 and 4). In primiparous cows, the culling risk was higer for cows in herds with higher first insemination conception rate (HR = 1.01, 95% CI 1.01; 1.02) (Table 3). On average, for every 500 kg increase in the herd’s average milk yield, the culling hazard was 4% higher (HR = 1.04, 95% CI 1.02; 1.06) in multiparous cows (Table 4).

The graphical assessment confirmed meeting the proportional hazard assumption of tested categorical variables in a log cumulative hazard plot as well as the overall fit of the models assessed by plotting Cox-Snell residuals against the cumulative hazards of individual observations at their failure times.

## Discussion

### Culling rates and farmers’ stated reasons for culling

There is no single optimal culling rate that is applicable to all herds for all years due to a variety of economic factors, farm capacities, individual cow factors, morbidity and mortality rates within the herd, availability of replacements, biosecurity considerations, etc. [1]. Not all studies have presented the culling rates due to death/slaughter excluding sales. In the current study, the average culling rate of Estonian dairy cows was 26.24 per 100 animal-years, excluding selling from the culling definition. In selected regions of the United States, the average culling rate was 31.6% in 1999 [3] but was 27.7% in Pennsylvanian herds in 2005 [21]. In Canada, Haine et al. [22] reported an average culling rate of 32% over the 2001–2010 decade and a dairy sell rate by 60 days in milk of 3.2%. The average culling rate of cows due to slaughter/death was 25.4%, ranging between 23% (in 2007) to 28% (in 2010) in Dutch dairy herds [23]. This shows that, on average, culling rates of the Estonian dairy cow population are mostly comparable to that reported in other countries.

Farmers’ stated reasons for culling were analysed in the present study due to the absence of more sound representative data. Care is needed when interpreting this data due to somewhat overlapping categories. Some cows might also have more than one reason for culling, whereas the ELPR system allows farmers to mark only one reason for each culling event. Also, the stated culling reasons might be the consequences of the primary disorder that might differ from what was reported by the farmers. Therefore these results are rather indicative and further studies including necropsy, meat inspection and laboratory data together with animal and disease history could reveal more reliable results. The four most common reasons for culling due to death and slaughter were: “feet/claw disorders”, “udder disorders”, “metabolic and digestive disorders”, and “fertility problems”, the order of frequency being broadly similar to those described by other authors [24]. The proportion of “feet/claw disorders” as the reason of culling was somewhat more prevalent compared to what was reported in studies performed in other European countries [6, 25]. As small herds with less than 20 cow-years were excluded in this study and majority of the Estonian dairy cows were housed in large herds [26], the conditions of large dairy herds and their effect on the cow hoof health were probably over-represented in the present study compared to other studies. In large Estonian dairy farms, cows are mostly housed in freestalls, and the latter is shown to be associated with an increased risk for lameness relative to other housing systems, including tie stalls and straw yards [27]. Additionally, other factors accompanying freestalls such as prolonged standing time due to milking [28], overstocking [29] or poor stall design [28] can increase the risk of lameness.

Metabolic disorders were a more common reason for culling in multiparous compared to primiparous cows. Roberts et al. [30] concluded that primiparous cows may have a different physiological response to post-calving metabolic challenges. The need to balance between energy demands for growth and milk production may have an effect on more effective fat mobilization before health and productivity are compromised [30].

A minor increase in the culling hazard occurred about a year after calving. As fertility problems were the primary reason for culling in the late lactation stage (≥ 200 DAC), we might assume that at that time farmers mostly cull their non-pregnant cows. Cows sent for slaughter should not be in their last trimester of pregnancy, according to a Motion for a European Parliament resolution [31]. The Estonian milk recording register system allows farmers to report only one reason for each culling event. Therefore it might be possible that some proportion of cows that were culled due to other reasons than fertility during the last third of lactation were also non-pregnant due to suffering from chronic health disorders or according to the farmers’ decisions.

Loss of a cow at its first lactation is economically most devastating and therefore undesirable for the dairy farmer [1]. In order to lower the culling rate of primiparous cows, measures promoting the good health of feet and udder are of utmost importance. In primiparous cows, dystocia and low milk yield both constituted roughly 9% of all culling reasons during the first 100 DAC, being nearly twice as high as that reported in multiparous cows. According to Mee [32], the feto-pelvic disproportion is the predominating risk factor for dystocia in primiparous cows, therefore bull selection as well as heifer nutrition and development might be critical factors lowering dystocia that leads to culling in primiparous cows.

We also identified some differences in culling reasons over lactations. Fertility, as a reason for culling, decreased in importance with each parity, indicating that probably more resilient cows in terms of breeding capability remained in the herds. Metabolic and digestive disorders as well as udder disorders were more frequently stated as the reason for culling in older cows. The fact that each calving event cumulatively adds the risk of suffering postpartum diseases, such as mastitis and ketosis [33, 34], might also explain this identified association.

### Animal-level risk factors for culling

In the current study, several common risk factors for culling in primiparous and multiparous cows were identified. Holstein breed cows had a significantly higher culling hazard compared to Estonian Red and Estonian Native breed cows. Holstein breed cows have a higher milk yield in Estonia [11]. Concomitantly, pure Holstein breed cows are more susceptible to production-related diseases [35] and have poorer reproductive performance than crossbred cows [36], thus being more prone to culling.

We identified that higher individual milk yield breeding value was a protective factor for culling, which may be explained by farmers trying to keep cows with good genetic merit. A milk yield breeding value could be calculated for cows who have at least two test-milking results available and whose sire has obtained a milk yield breeding value. The category “missing” also included cows that were culled during early lactation, which might be the cause of high culling hazard among primiparous cows who had no breeding value in the dataset.

In agreement with other studies, it is essential to pay attention to predisposing conditions and transmission of infectious diseases that might be associated with the incidence of stillbirth or abortion, as these are important risk factors for cow culling and longevity [37, 38]. Additionally, higher culling risk due to giving birth to a male calf compared to a female calf indicates that birth weight of an offspring might be an important factor in terms of culling via increasing the probability for dystocia. This is known to be associated with a higher mortality hazard in cows [33] as well as an increased risk of post-partum diseases eventually leading to culling [39]. Furthermore, calving for the first time at a higher age was associated with a higher culling hazard. Interestingly, the association was also present in multiparous cows, suggesting a possible long-term impact. Heifers might calve at an older age due to management factors, health disorders, feeding management, or due to herd breeding strategies [23, 40], and the reason for the old age at first calving may be more important for the culling risk than the age per se.

In multiparous cows, a longer previous calving interval was associated with a higher culling probability at next lactation. Prolonged calving interval might be related to negative energy balance and diseases associated with the early post-partum period, delaying conception. Due to the possible recurrent propensity of post-partum diseases at the cow level, the undesired impact of a longer calving interval might manifest at the next lactation. In addition, a longer calving interval allows cows to gain more weight, which may be a risk factor for developing post-partum diseases, increasing the culling hazard [41].

High somatic cell count at last test-milking during the previous lactation and/or at first test-milking after calving, indicating the presence of subclinical intramammary infection, was a factor associated with higher culling risk during lactation. The presence of clinical or subclinical mastitis is a known risk factor for dairy cow culling [39]. Lower milk yield at the end of the previous lactation or at the first test-milking of ongoing lactation was also associated with a higher culling probability. It has been shown that, in general, farmers are more eager to cull low-producing cows [39]. Still, low milk yield soon after calving might be associated with an underlying disease. The high milk fat/protein ratio is a valuable indicator of ketosis in the early post-partum period [42], and it is concomitantly related to increased probabilities of developing displaced abomasum, retained placenta, metritis, clinical endometritis and clinical ketosis as well as higher culling probabilities [43, 44].

### Herd-level factors associated with culling

On average, the cow culling hazard was higher in larger herds. The association between herd size and the health and welfare of dairy cows is complex and includes the impact of several factors, including facilities, management and operational factors [20, 45]. According to previous studies, the positive association between the incidence of metabolic diseases and herd size have been found [46]. Also, the presence and dynamics of infectious diseases and different biosecurity management could explain higher culling risk in larger herds [23]. According to Gieseke and co-authors [47], housing conditions and management practices have a greater effect on cow welfare than the herd size itself and more research is needed to identify factors in large farms that affect animal health. Simultaneously to the European Union, the milk price dropped suddenly in the autumn of 2014 in Estonia [48], resulting in a 5.2 and 11% reduction of the number of dairy cows and herds, respectively, in Estonia during the year of 2015 [49]. Due to this, change of herd size was controlled as a factor in the statistical models to account for its confounding effect.

Higher herd average milk yield was associated with increased culling hazard of individual cows in our study. Several previous studies have found that higher milk yield has an adverse effect on the cows’ resistance to diseases, as it is correlated with presence of the clinical mastitis, reproductive diseases [50], and other postpartum disorders [43]. Although high milk yield and the high genetic potential for milk production are often been blamed for the short longevity of dairy cows, this does not always seem to affect cow longevity [51]. On the other hand, due to uncertain causality, herds with higher milk yield might also have better reproductive performance, allowing more cows to be culled.

The current study showed that herds with a longer average calving interval had a lower risk of culling. A longer calving interval may be the consequence of fertility problems in the herd or the result of a voluntary decision of the farmer to delay with breeding after calving and thus to extend the lactation period [52]. Although a shorter calving interval is considered as economically optimal [53], an economic benefit in extending lactations in high-yielding cows was also found [54]. In the study by Allore and Erb [55], a lower risk of culling for reproductive failure was present in herds with an extended voluntary waiting period. Still, due to a cross-sectional study design, it is impossible to draw causal inferences, and the identified association might result from farm lower thresholds for culling non-pregnant cows [39].

In the current study, the cow culling hazard was also negatively associated with herd average lactation number that cumulatively aggregates the individual animal culling hazards.

Using robot milking system was a protective factor for culling compared to other milking methods. Additionally, cows that were milked three times a day at first test-milking had a lower culling probability throughout the lactation compared to cows that were milked twice a day. Farms with automatic milking systems differ from those with other milking systems in many aspects, such as environmental conditions, feeding management, grouping policies, etc. Unfortunately, it was not possible to discriminate milking method, but only the milking frequency, meaning that the identified associations could be affected by other factors.

### Validity and limitations of the study

The present study included lactation-level records of all cows from all herds that had at least 20 cow-years in years 2013–2015 in Estonia and participated in the milk recording system. According to the ELPR [26], 94.1% (in 2013) to 95.4% (in years 2014 and 2015) of the Estonian dairy cow population was enrolled in the milk recording system, probably leaving out smaller farms that produced milk for their own consumption. After skipping herds with < 20 cow-years, we emphasize a good external validity of this study for medium or large sized herds, whereas the study results should not be extrapolated to small dairy holdings.

When analysing farmers’ reported reasons for culling, a reporting bias might be present. In Estonia, farmers are allowed to report only one reason for each animal exit to the milk recording register. Still, studies have shown that in many instances, farmers report more than one reason of culling when allowed [56, 57]. To our knowledge, there are no studies that investigate the farmers’ behaviour in reporting culling reasons. We assume that farmers report the main and most obvious reason of culling at the time the cow is leaving the herd. However, this might not be the primary or ultimate disease or disorder which leads to culling.

In the EU, there is a harmonized mandatory registration and reporting of animal births, movements, and deaths [58] making registry data reliable for research purposes. As participation in the milk recording register is voluntary, the reporting in that system might not always be precise. Although the animal registry and milk recording registry makes crosschecks in their data, small discrepancies were found when comparing the cows’ exit dates in the two registries. Also, some cows had very long lactations and low number of culls occurred far away from the latest calving (Fig. 2) in which a new calving date might have remained unreported in the registry. Still, due to the high sample size of this study, these aberrations probably have no effect on the overall results and conclusions.

## Conclusions

Culling rates of Estonian dairy cows housed in farms with more than 20 cows were comparable to that reported in other countries. More attention should be paid to prevention of feet/claw disorders and mastitis, because they accounted for roughly half of the reported culling reasons. The early post-partum period was the period bearing the highest risk for cow culling, with metabolic diseases and feet/claw diseases being the most frequently stated reasons for culling at that time. Our analyses confirmed the importance of easy calving and ensuring good health around calving to avoid the loss of a cow. Due to the intensification of dairy production and the identified higher culling hazards in larger herds, further studies should concentrate on factors that contribute to cow culling and longevity in large commercial herds. Along these same lines, a widened list of herd factors, including animal housing, environment, management as well as farm workers’ attitude and motivation, should be studied helping to understand the complex problem of cow culling and combat the problem of reduced cow longevity.

## Methods

### Datasets

All data used for the present study was based on recordings in the Estonian Farm Animal Register kept by the Estonian Agricultural Registers and Information Board and data of Estonian Livestock Performance Recording Ltd. (**ELPR**, national voluntary animal production system involved with monthly test-milkings including data of around 95% of dairy cows in Estonia, called “milk recording register” hereafter). Cow lactation level records for the period between January 1, 2013 and December 31, 2015 were collected from the milk recording register. Data was required of all Estonian dairy herds that participated in the milk recording register and had a herd size of at least 20 cows at the start of the study period. The initial datasets included lactation level data of 86,459 primiparous and 109,314 multiparous cows (177,712 lactations) from 409 and 410 dairy herds, respectively.

The lactation level observation period started at the day of calving and ended at the date the cow left the herd, had a new calving, or up to the end of the study period (December 31, 2015). Due to the aim of reducing the impact of voluntary culling in the analysis, the definition “culling” established by [1] was modified in the sense that sold cows (cows that were sold to another farm for productive life) were considered as right censored observations, thus excluding sales from the culling definition. Therefore the definition of culling in this study includes on-farm mortality (unassisted death and euthanasia) and slaughter (sending cows to an abattoir).

Farmers are obliged to report all movements and exits of cattle to the Estonian Farm Animal Register within 7 days [59]. In the Farm Animal Register, it is specified for each exit event whether the animal died on-farm, was euthanized, got lost, was slaughtered or was sold/exported. Farmers report the reason of exit in the milk recording register, choosing one reason from the list of 24 pre-defined categories, including selling, old age, low milk yield, mastitis, metabolic disorders, abortion, etc. (the complete list is given below in the section “Data editing”). The definition of these categories was not provided to the farmers by the registry. In order to analyse the proportion of deaths (including euthanasia) and slaughter as well as include a reason for each exit event, the data of two registries was merged by cow identification numbers. Due to the fact that the analyzed datasets were on a lactation level and Farm Animal Register data on cow level and one animal might have more than one exit during its lifetime (e.g., sold and slaughtered/died), the exit dates of the two datasets were compared in order to connect a correct exit type (death / euthanasia / selling / slaughter) and farmers’ stated reason for animal exit. Reporting reasons of cattle exit in the milk recording register by farmers is voluntary, therefore it was expected that the dates of cattle exit in two datasets would slightly differ. When joining the datasets of two registries, differences of exit dates up to seven days were allowed and were considered to be the same exit event.

The data from Farm Animal Register consisted of farm identification number together with the owner’s name, location of a herd, animal identification number, birth date, sex and breed of an animal, exit date and type of exit. Milk recording register data included animal, lactation and herd level data. A more detailed description of the data provided in the milk recording system is described in [60].

### Data editing

Due to inexplicable short calving intervals (calving interval < 290 days and no stated abortion or stillbirth in the registry), 86 and 151 lactation records were removed from the datasets of primiparous and multiparous cows, respectively.

Ten categories of culling reasons were created by compiling together similar culling reasons available in ELPR system. Farmers’ stated reasons for culling due to death and slaughter were categorized as follows:

- feet and claw disorders: included pre-defined categories “undesirable leg conformation”, “leg traumas” and “leg disorders”;
- respiratory and infectious diseases: “respiratory diseases” and “infectious diseases”;
- metabolic and digestive disorders: “metabolic disorders”, “milk fever” and “gastrointestinal disorders”;
- fertility problems: “fertility problems”, “gynecological diseases”, “abortion”;
- dystocia;
- trauma and accident: “other traumas” and “accident”;
- udder disorders: “udder flaws”, “udder and teat traumas” and “mastitis”;
- low milk yield;
- age;
- other reasons: “animal lost”, “bad temperament”, “bad milking”, “selling” and “other reasons”.

The category “other reasons” included farmers’ stated reasons that were rarely reported or not reasonably associated with death and slaughter, thus considered to be recording mistakes. In order to determine whether the cow was purchased or not, the birth farm identification number was compared with the farm number in which the cow was located. Milk yield breeding value was not available for all cows (in case the sire of a cow had not received a milk yield breeding value or the cow had less than two test milking results available), thus the variable was categorised into roughly equally sized groups with a separate category for cows with missing information. Due to a low number of Estonian Native breed cows, these observations were merged with Estonian Red breed cows. Counties were combined into four regions: Northeast, Southeast, Southwest, and Northwest regions. Four seasons were created based on the calving dates of the cows: winter (December–February), spring (March–May), summer (June–August) and autumn (September–November). The variable “change of herd size” was created by comparing the number of cows in the herd between 2013 to 2015. Based on that, five categories were created: no considerable change in the number of cows (± 5%), decrease > 5 to 15%, decrease > 15%, increase > 5 to 15% and increase > 15%.

Data related with test-milkings was categorized when a biological threshold for discriminating normal from pathological conditions was available. The variable “milk somatic cell count” had two categories separated at the level of 200,000 cells/ml [61]. A threshold of 1.5 was used to dichotomise “milk fat/protein ratio” [43] and 6.78 mmol/L was the limit value for “milk urea content” [62].

For the herd-level production and reproduction data, three-year averages were calculated (Supplementary Table 1A).

### Data analysis

A descriptive statistical data analysis was performed to identify farmers´ stated reasons for culling. The analysis was performed separately per parity as well as by stage of lactation.

Risk factor analysis for cow culling (on-farm death, euthanasia or slaughter) was performed separately for primiparous and multiparous cows due to different sets of variables used. A complete description of the statistical methods used can be found in [60]. Briefly explained, the Weibull proportional hazard regression model specifying herd as a random factor (gamma distributed frailty effect) was applied specifying culling as a failure event. Lactation level observation period started at the day of calving or at the day of purchase of a lactating cow. In order to account for the left truncation in the analyses [63] the “start date” was accounted as January 1, 2013 for observations that started before that date by specifying the “stset” options in STATA MP 14 software (StataCorp LP, College Station, USA). Observations that ended with new calving, selling, or the end of the study period were right censored. Due to several lactations of multiparous cows in the dataset during a study period, the animal identification number was specified as the multiple-record ID variable when setting time-to-event data. A Kaplan-Meier survival curve was created to compare the survival probability of primiparous and multiparous cows over lactation by plotting estimates survival curves. Culling rates together with 95% confidence intervals were calculated by using the Mantel–Haenszel method. Estimated cumulative survivor hazard function was calculated to describe the culling probabilities at different time-points over lactation. In order to identify the difference of culling hazards between primiparous and multiparous cows, a univariable proportional hazard Weibull regression model was composed including cow parity (primiparous / multiparous) as a fixed effect and herd as a frailty term.

After univariable screening of the predictors in their association with culling probability all variables with a liberal *p*-value of < 0.05 and not collinear to each other were considered as candidates in multivariable analysis. A multivariable Weibull model was built by removing insignificant predictor variables from the model by manual backward elimination technique. Due to the variable “Assisted calving” having a time-dependent effect, the lactation-level observation period was split on the seventh day post-calving using the ‘stsplit’ command. Due to the high power of the analyses coming from a large sample size, the predictor variables included in the final model had to be associated with the culling hazard at a 1% significance level.

Due to differences in the beginning of the observation period, a separate model was conducted to analyse the associations between first test-milking results and culling during lactation controlling for confounding variables (“days in milk”, “breed”, “parity”, “calving year”, “herd average number of cows” and “change of herd size from 2013 to 2015”) in the model. In that model, the observation period started from the day of the first test-milking and lasted until culling (failure event), right censoring due to new calving, selling of an animal or the end of the study period. The model included the data of primiparous and multiparous cows.

Akaike’s and Bayesian Information Criteria (**AIC** and **BIC**, respectively) values were used for comparing models, and lower AIC/BIC values determined the better model. Plots of the cumulative hazard versus Cox-Snell residuals were generated to assess the model fit. Proportional hazard assumption was evaluated by graphical assessment of log-cumulative hazard plots [64]. Statistical analyses were performed using STATA MP version 14 (StataCorp LP, College Station, USA).

## Supplementary information

**Additional file 1 Supplementary Table 1A.** Descriptive statistics of potential continuous risk factors for culling of 86,373 primiparous and 109,295 multiparous dairy cows with 177,561 lactations in Estonia between January 1, 2013 and December 31, 2015.

**Additional file 2 Supplementary Table 1B**. Descriptive statistics of potential categorical risk factors for culling of 86,373 primiparous and 109,295 multiparous dairy cows with 177,561 lactations in Estonia between January 1, 2013 and December 31, 2015.

## Data Availability

Access to data used in present study was provided by the Estonian Farm Animal Register kept by the Estonian Agricultural Registers and Information Board and Estonian Livestock Performance Recording Ltd., according to agreement. Restrictions apply to the availability of these data and so are not publicly available.

## Abbreviations

CR: Culling rate
HR: Hazard rate
DAC: Days after calving
SCC: Somatic cell count
ELPR: Estonian Livestock Performance Recording Ltd.
AIC: Akaike’s Information Criteria
BIC: Bayesian Information Criteria
