# Supplementary material for: Culling reasons and risk factors in Estonian dairy cows
Source: BMC Vet Res. 2020 Jun 1;16:173. doi: 10.1186/s12917-020-02384-6 (PMC7268674; doi:10.1186/s12917-020-02384-6)
Supplement: Supplementary file 1 — Additional file 1 Supplementary Table 1A. Descriptive statistics of potential continuous risk factors for culling of 86,373 primiparous and 109,295 multiparous dairy cows with 177,561 lactations in Estonia between January 1, 2013 and December 31, 2015. [file 12917_2020_2384_MOESM1_ESM.docx]

| **Supplementary table 1A.** Descriptive statistics of potential continuous risk factors for culling of 86,373 primiparous and 109,295 multiparous dairy cows with 177,561 lactations in Estonia between January 1, 2013 and December 31, 2015 | | | | | | | | |
| --- | --- | --- | --- | --- | --- | --- | --- | --- |
|  | Primiparous cows | | | | Multiparous cows | | | |
| Variable | median | quartiles | missing obs (n^1,2,3^) | p-value^d^ | median | quartiles | missing obs (n^1,2,3^) | P-value^4^ |
| *Animal level variables* |  |  |  |  |  |  |  |  |
| Age at first calving (months)^1^ | 25.9 | 24.2; 28.6 | 25 | <0.001 | 26.5 | 24.7; 29.2 | 19 | <0.001 |
| Length of previous calving interval (days)^2^ |  |  |  |  | 393.0 | 359.0; 450.0 | 894 | <0.001 |
| Length of dry period in previous lactation (days)^2^ |  |  |  |  | 62.0 | 53.0; 75.0 | 3 | <0.001 |
| Days in milk at last test-milking in previous lactation^2^ |  |  |  |  | 312.0 | 280.0; 363.0 | 0 | <0.001 |
| Milk yield at last test-milking in previous lactation (kg)^2^ |  |  |  |  | 15.6 | 11.3; 20.2 | 1,721 | <0.001 |
| Days in milk at first milk-testing^2^ | 21.0 | 13.0;28.0 | 4,878 | <0.001 | 21.0 | 13.0; 28.0 | 14,255 | <0.001 |
| Milk yield at first test-milking (kg)^2^ | 27.3 | 22.9; 31.7 | 5,842 | <0.001 | 36.1 | 29.6; 42.4 | 16,835 | <0.001 |
| *Herd level variables* |  |  |  |  |  |  |  |  |
| Number of cows^3^ | 90.3 | 39.9; 256.0 | 0 | <0.001 | 89.5 | 37.7; 256.0 | 0 | <0.001 |
| Herd average milk yield (kg per cow per year)^3^ | 7711.0 | 6107.7; 8874.3 | 0 | <0.001 | 7710.7 | 6107.3; 8874.3 | 0 | <0.001 |
| Herd average milk fat/protein ratio^3^ | 1.2 | 1,2: 1.3 | 0 | <0.001 | 1.2 | 1.2; 1.3 | 0 | <0.001 |
| Herd average milk somatic cell count (*1000/mL)^3^ | 326.0 | 250.0; 425.3 | 0 | 0.002 | 325.0 | 250.0; 425.3 | 0 | 0.014 |
| Herd average milk urea (mg/L)^3^ | 240.0 | 221.0; 258.3 | 0 | 0.425 | 240.2 | 221.0; 258.3 | 0 | 0.264 |
| Herd average age at first calving (months)^3^ | 27.9 | 26.2; 30.6 | 0 | <0.001 | 27.9 | 26.2; 30.7 | 0 | <0.001 |
| Herd average calving interval (days)^3^ | 422.7 | 406.3; 448.3 | 0 | 0.005 | 422.5 | 406.3; 448.3 | 0 | 0.035 |
| Herd average lenght of dry period (days)^3^ | 69.7 | 64.0; 77.7 | 0 | 0.001 | 69.8 | 64.0; 78.0 | 0 | <0.001 |
| Herd average interval from calving to insemination (days)^3^ | 97.0 | 84.3; 119.3 | 19 | 0.001 | 97.0 | 84.3; 119.3 | 20 | <0.001 |
| Herd average calving to conception interval (days)^3^ | 143.0 | 127.3; 170.7 | 20 | 0.012 | 143.0 | 127.3; 170.7 | 21 | 0.055 |
| Herd average number of inseminations per conception^3^ | 1.9 | 1.6; 2.2 | 19 | 0.039 | 1.9 | 1.6; 2.2 | 20 | <0.001 |
| Herd average first insemination conception rate (%)^3^ | 54.0 | 46.8; 60.9 | 18 | 0.381 | 54.0 | 46.8; 60.9 | 19 | 0.181 |
| Herd average number of lactations^3^ | 2.5 | 2.3; 2.9 | 0 | <0.001 | 2.5 | 2.3; 2.9 | 0 | <0.001 |
| Herd average age at first insemination in heifers (months)^3^ | 17.4 | 15.7; 20.5 | 53 | 0.006 | 17.4 | 15.7; 20.5 | 54 | <0.001 |
| Herd average proportion of stillbirths in (%)^3^ | 6.9 | 4.9; 9.1 | 0 | 0.022 | 6.9 | 5.0; 9.2 | 0 | 0.003 |
| Herd average proportion of abortions (%)^3^ | 0.8 | 0.0; 1.6 | 0 | 0.016 | 0.8 | 0.0; 1.6 | 0 | <0.001 |
| ^1^number of cows |  |  |  |  |  |  |  |  |
| ^2^number of observations |  |  |  |  |  |  |  |  |
| ^3^number of herds |  |  |  |  |  |  |  |  |
| ^4^identified in an univariable Weibull proportional hazard random effect model (herd as random effect) | | | | | | | | |
