# Supplementary material for: Culling reasons and risk factors in Estonian dairy cows
Source: BMC Vet Res. 2020 Jun 1;16:173. doi: 10.1186/s12917-020-02384-6 (PMC7268674; doi:10.1186/s12917-020-02384-6)
Supplement: Supplementary file 2 — Additional file 2 Supplementary Table 1B. Descriptive statistics of potential categorical risk factors for culling of 86,373 primiparous and 109,295 multiparous dairy cows with 177,561 lactations in Estonia between January 1, 2013 and December 31, 2015. [file 12917_2020_2384_MOESM2_ESM.docx]

| **Supplementary table 1B**. Descriptive statistics of potential categorical risk factors for culling of 86,373 primiparous and 109,295 multiparous dairy cows with 177,561 lactations in Estonia between January 1, 2013 and December 31, 2015 | | | | | | | |
| --- | --- | --- | --- | --- | --- | --- | --- |
|  |  | Primiparous cows | | | Multiparous cows | | |
| Variable | Category | N^1,3^ | CR^4^ | p-value^5^ | N^1,2,3^ | CR^4^ | p-value^5^ |
| *Animal level variables* |  |  |  |  |  |  |  |
| Purchased (born in other herd)^2^ | No | 79,306 | 15.97 | 0.159 | 154,630 | 31.71 | <0.001 |
|  | Yes | 7,067 | 15.73 |  | 22,931 | 32.67 |  |
| Breed^1^ | Estonian Holstein | 70,959 | 16.61 | <0.001 | 87,053 | 33.49 | <0.001 |
|  | Estonian Red and Estonian Native | 15,414 | 12.92 |  | 22,242 | 25.75 |  |
| Milk yield breeding value^1^ | <90 | 17,784 | 16.35 | <0.001 | 24,150 | 38.50 | <0.001 |
|  | 90-96 | 20,073 | 11.19 |  | 23,931 | 33.88 |  |
|  | 97-104 | 18,088 | 8.42 |  | 27,621 | 30.70 |  |
|  | ≥105 | 21,325 | 6.81 |  | 27,976 | 27.18 |  |
|  | Not estimated | 9,103 | NE^6^ |  | 5,617 | 22.15 |  |
| Calving year^2^ | 2013 | 28,087 | 15.69 | 0.027 | 57,942 | 30.43 | <0.001 |
|  | 2014 | 29,600 | 14.95 |  | 60,021 | 31.52 |  |
|  | 2015 | 28,686 | 18.76 |  | 59,598 | 35.51 |  |
| Abortion in previous lactation^2^ | No |  |  |  | 176,714 | 31.80 | 0.005 |
|  | Yes |  |  |  | 847 | 38.94 |  |
| Breeding^2^ | Using a bull | 23,585 | 17.11 | <0.001 |  |  |  |
|  | AI, 1 insemination per pregnancy | 38,101 | 14.89 |  |  |  |  |
|  | AI, 2 inseminations per pregnancy | 14,146 | 16.18 |  |  |  |  |
|  | AI, ≥3 inseminations per pregnancy | 7,690 | 18.04 |  |  |  |  |
|  | Missing | 2,851 | 13.66 |  |  |  |  |
| Season at calving^2^ | Winter (Dec, Jan, Feb) | 22,094 | 16.03 | 0.001 | 43,965 | 32.14 | <0.001 |
|  | Spring (March, Apr, May) | 22,549 | 16.31 |  | 42,150 | 30.91 |  |
|  | Summer (June, July, Aug) | 21,074 | 16.34 |  | 49,895 | 32.45 |  |
|  | Autumn (Sept, Oct, Nov) | 20,656 | 14.93 |  | 41,551 | 31.82 |  |
| Parity^2^ | First | 86,373 | 15.95 |  |  |  |  |
|  | Second |  |  |  | 67,349 | 20.94 | <0.001 |
|  | Third |  |  |  | 47,157 | 30.75 |  |
|  | Fourth |  |  |  | 30,225 | 40.40 |  |
|  | Fifth |  |  |  | 17,319 | 48.57 |  |
|  | Sixth |  |  |  | 8,821 | 53.56 |  |
|  | ≥Seventh |  |  |  | 6,690 | 61.82 |  |
| Calf^2^ | Female | 38,752 | 13.87 | <0.001 | 76,647 | 29.07 | <0.001 |
|  | Male | 36,763 | 15.66 |  | 83,784 | 30.91 |  |
|  | Twins/triplets | 488 | 20.89 |  | 6,793 | 43.69 |  |
|  | Stillbirth | 10,034 | 25.61 |  | 9,651 | 55.17 |  |
|  | Abortion | 331 | 15.26 |  | 686 | 81.36 |  |
|  | Missing | 5 | NE^6^ |  | 0 | NE^6^ |  |
| Stillbirth^2^ | No | 76,334 | 14.78 | <0.001 | 167,910 | 30.68 | <0.001 |
|  | Yes | 10,034 | 25.61 |  | 9,651 | 55.17 |  |
|  | Missing | 5 | NE^6^ |  | 0 | NE^6^ |  |
| Abortion^2^ | No | 86,037 | 15.95 | 0.797 | 176,875 | 31.70 | <0.001 |
|  | Yes | 331 | 15.26 |  | 686 | 81.36 |  |
|  | Missing | 5 | NE^6^ |  |  |  |  |
| Assisted calving^2^ | No | 70,907 | 14.90 | <0.001 | 162,580 | 31.16 | <0.001 |
|  | Yes | 15,466 | 20.87 |  | 14,981 | 39.32 |  |
| Milk fat/protein ratio at last test-milking in previous lactation^2^ | <1.5 |  |  |  | 165,703 | 31.58 | 0.524 |
|  | ≥1.5 |  |  |  | 8,388 | 30.21 |  |
|  | Missing |  |  |  | 3,470 | NE^6^ |  |
| Milk somatic cell count at last test-milking in previous lactation ( *1000/mL)^2^ | <200 |  |  |  | 96,074 | 25.51 | <0.001 |
|  | ≥200 |  |  |  | 78,847 | 39.50 |  |
|  | Missing |  |  |  | 2,640 | NE^6^ |  |
| Milk urea content at last test-milking in previous lactation (mg/dL)^2^ | <19 |  |  |  | 43,443 | 29.91 | 0.002 |
|  | ≥19 |  |  |  | 131,210 | 32.10 |  |
|  | Missing |  |  |  | 2,908 | NE^6^ |  |
| Milk fat/protein ratio at first test-milking^2^ | <1.5 | 62,093 | 11.06 | <0.001 | 119,581 | 21.85 | <0.001 |
|  | ≥1.5 | 15,459 | 15.57 |  | 35,567 | 31.70 |  |
|  | Missing | 4,038 | NE^6^ |  | 8,179 | NE^6^ |  |
|  | Left censored before first test-milking | 4,783 | NE^6^ |  | 14,234 | NE^6^ |  |
| Milk somatic cell count at first test-milking ( *1000/mL)^2^ | <200 | 61,061 | 10.55 | <0.001 | 114,272 | 21.05 | <0.001 |
|  | ≥200 | 16,866 | 17.70 |  | 41,759 | 33.38 |  |
|  | Missing | 3,663 | NE^6^ |  | 7,296 | NE^6^ |  |
|  | Left censored before first test-milking | 4,783 | NE^6^ |  | 14,234 | NE^6^ |  |
| Milk urea content at first test-milking (mg/dL)^2^ | <19 | 27,814 | 12.75 | <0.001 | 63,837 | 24.79 | <0.001 |
|  | ≥19 | 50,078 | 11.65 |  | 92,056 | 23.70 |  |
|  | Missing | 3,698 | NE^6^ |  | 7,434 | NE^6^ |  |
|  | Left censored before first test-milking | 4,783 | NE^6^ |  | 14,234 | NE^6^ |  |
| *Herd level variables* |  |  |  |  |  |  |  |
| Milking method at last test-milking in previous lactation^2^ | Milking twice a day |  |  |  | 138,392 | 32.78 | <0.001 |
|  | Milking three times a day |  |  |  | 16,359 | 28.94 |  |
|  | Robot milking |  |  |  | 22,662 | 27.90 |  |
|  | Missing |  |  |  | 148 | NE^6^ |  |
| Milking method at first test-milking^2^ | Milking twice a day | 56,391 | 13.66 | <0.001 | 111,403 | 26.36 | <0.001 |
|  | Milking three times a day | 13,808 | 11.08 |  | 28,631 | 23.42 |  |
|  | Robot milking | 9,201 | 8.22 |  | 19,181 | 18.91 |  |
|  | Missing | 2,190 | NE^6^ |  | 4,112 | NE^6^ |  |
|  | Left censored before first test-milking | 4,783 | NE^6^ |  | 14,234 | NE^6^ |  |
| Change of herd size from 2013 to 2015^3^ | No change (±5%) | 135 | 16.17 | <0.001 | 135 | 32.79 | <0.001 |
| Decrease >5 to 15% | 71 | 18.15 |  | 71 | 31.96 |  |  |
| Decrease >15% | 67 | 19.85 |  | 68 | 33.51 |  |  |
|  | Increase >5 to 15% | 74 | 12.71 |  | 74 | 29.00 |  |
|  | Increase >15% | 62 | 13.62 |  | 62 | 30.35 |  |
| Region^3,7^ | Northeast | 114 | 18.36 | 0.009 | 114 | 36.84 | <0.001 |
|  | Southeast | 95 | 14.23 |  | 98 | 28.15 |  |
|  | Southwest | 120 | 13.20 |  | 120 | 26.23 |  |
|  | Northwest | 79 | 15.53 |  | 77 | 33.41 |  |
|  | Missing | 1 | NE^6^ |  | 1 | NE^6^ |  |
| ^1^number of cows | | | | | | | |
| ^2^number of observations | | | | | | | |
| ^3^number of herds | | | | | | | |
| ^4^culling rate (per 100 animal-years) | | | | | | | |
| ^5^identified in an univariable Weibull proportional hazard random effect model (herd as random effect) | | | | | | | |
| ^6^not estimated | | | | | | | |
| ^7^Northeast Estonia: Ida-Viru, Lääne-Viru, Jõgeva, Järva county; Southeast Estonia: Tartu, Valga, Võru, Põlva county; Southwest Estonia: Pärnu, Viljandi, Saare county; Northwest Estonia: Harju, Rapla, Lääne, Hiiu county | | | | | | | |
